# Longitudinal Trajectories of Hair Cortisol: Hypothalamic-Pituitary-Adrenal Axis Dysfunction in Early Childhood

**DOI:** 10.3389/fped.2021.740343

**Published:** 2021-10-11

**Authors:** Cynthia R. Rovnaghi, Joseph Rigdon, Jean-Michel Roué, Monica O. Ruiz, Victor G. Carrion, Kanwaljeet J. S. Anand

**Affiliations:** ^1^Pain/Stress Neurobiology Lab, Maternal and Child Health Research Institute, Stanford University School of Medicine, Stanford, CA, United States; ^2^Quantitative Sciences Unit, Stanford University School of Medicine, Stanford, CA, United States; ^3^Department of Pediatrics, University Hospital of Brest, Brest, France; ^4^Laboratory LIEN, University of Brest, Brest, France; ^5^Department of Pediatrics, Stanford University School of Medicine, Stanford, CA, United States; ^6^Department of Psychiatry and Behavioral Sciences, Stanford University School of Medicine, Stanford, CA, United States

**Keywords:** hair cortisol concentrations, chronic stress, child development, cortisol trajectories, hypothalamic-pituitary-adrenal axis, HPA axis dysregulation

## Abstract

The objective of this study was to examine if longitudinal trajectories of hair cortisol concentrations (HCC) measured at two or three yearly time points can identify 1-3 year old children at risk for altered hypothalamic-pituitary-adrenal (HPA)-axis function due to early life stress (ELS). HCC was measured (*N* = 575) in 265 children using a validated enzyme-linked immunosorbent assay. Hair was sampled in Clinic Visits (CV) centered at years 1, 2, and 3 (*n* = 45); 1 and 2 (*n* = 98); 1 and 3 (*n* = 27); 2 and 3 (*n* = 95). Log-transformed HCC values were partitioned using latent class mixed models (LCMM) to minimize the Bayesian Information Criterion. Multivariable linear mixed effects models for ln-HCC as a function of fixed effects for age in months and random effects for participants (to account for repeated measures) were generated to identify the factors associated with class membership. Children in Class 1 (*n* = 69; 9% Black) evidenced declining ln-HCC across early childhood, whereas Class 2 members (*n* = 196; 43% Black) showed mixed trajectories. LCMM with only Class 2 members revealed Class 2A (*n* = 17, 82% Black) with sustained high ln-HCC and Class 2B (*n* = 179, 40% Blacks) with mixed ln-HCC profiles. Another LCMM limited to only Class 2B members revealed Class 2B1 (*n* = 65, 57% Black) with declining ln-HCC values (at higher ranges than Class 1), and Class 2B2 (*n* = 113, 30% Black) with sustained high ln-HCC values. Class 1 may represent hair cortisol trajectories associated with adaptive HPA-axis profiles, whereas 2A, 2B1, and 2B2 may represent allostatic load with dysregulated profiles of HPA-axis function in response to varying exposures to ELS. Sequential longitudinal hair cortisol measurements revealed the allostatic load associated with ELS and the potential for developing maladaptive or dysregulated HPA-axis function in early childhood.

## Introduction

The stress response forms a highly conserved regulatory system in all eukaryotic multicellular organisms, evolutionarily designed to cope with a broad range of stimuli that may threaten, or be perceived as threatening to their survival, growth, and/or dynamic equilibrium (“homeostasis”). The stress response includes the neuroendocrine, neuroimmune, and other systems and a central modulator of these systems is the hypothalamic-pituitary-adrenal (HPA)-axis (1). Allostatic load is defined as the cumulative burden of exposures to repeated or chronic stressors, associated with deleterious consequences for growth and survival as well as longterm changes in the stress response system (2). Chronic or repetitive exposures to early life stress (ELS) alter HPA-axis regulation, associated with the early onset of chronic non-communicable diseases (NCDs) and psychopathology (3, 4). The American Academy of Pediatrics (AAP) made an appeal for distinguishing adaptive, maladaptive, and toxic stress in children and declared that “*Identifying children at high risk for toxic stress is the first step in providing targeted support for their parents and other caregivers”* (5).

Cortisol is the hormonal end-product of the HPA-axis that regulates the endocrine and metabolic stress responses, brain and other organ development, and immune functions. Transitory changes in salivary or serum cortisol may reflect exposures to acute stress (6) but cannot capture cumulative exposures to chronic or repetitive stress. Cortisol binds to growing hair; thus, hair cortisol concentrations (HCC) can provide a summative measure of responses to stressful experiences over the previous 3-6 months (7, 8). A recent review proposed that HCC may represent a measure of recent/acute stress, but this meta-analysis included studies from 16 animal species, ignored major differences between animal fur and human scalp hair, and analyzed studies with cross-sectional rather than longitudinal data (9). HCC in humans are positively correlated with repeated measures of salivary cortisol (8).

Adverse childhood experiences (ACEs) alter subsequent stress responses associated with HPA-axis dysregulation, particularly among Black adults (10–12). A dysregulated HPA-axis often shows persistent hyper- or hypo-responsiveness, associated with a reduced cortisol awakening response, flattened diurnal cortisol slope, higher evening cortisol levels, and dampened responses to corticotrophin (ACTH) or corticotrophin-releasing hormone (CRH) (13). Preclinical data illustrate progressive phases of altered HPA-axis function ultimately leading to HPA-axis dysregulation. However, a substantial gap exists between preclinical and clinical data because longitudinal human studies have not mapped these phases of HPA-axis function, particularly among young children. Heightened neuroplasticity in children aged 0-3 years makes them vulnerable to long-term changes in the HPA-axis regulation following exposure(s) to ELS, linked epidemiologically with poor physical and mental health across the lifespan (1, 5, 13–15).

Our overarching goal is to measure the long-term effects of ELS on the HPA axis. We have previously published novel methods for measuring HCC (16) and socioeconomic adversity (17), and also identified the demographic and psychosocial factors related to HCC in a cross-sectional population of preschool children (18, 19). Black children showed higher HCC than White/other children because of exposure to psychosocial stressors and daily microaggressions; these differences persisted even after adjusting for socioeconomic factors. HCC values measured at earlier ages were significant predictors of HCC at later ages (18), therefore we sought to examine longitudinal HCC trajectories using latent class mixed models (LCMM). Longitudinal analyses may be more likely to identify individual children with altered states of HPA axis regulation during early childhood, and therefore, may be more vulnerable to the longterm effects of ELS on the subsequent cognitive, behavioral, educational, psychosocial, physical health and psychiatric outcomes. If these children can be identified, they would be eligible for potential therapeutic interventions to prevent or ameliorate these longterm effects.

Compared to cluster analyses, structural equation models, or generalized estimating equation models, LCMM is more suitable for investigating developmental trajectories because it is robust to missing values and categorical variables, and is specifically designed to partition groups of individuals with similar profiles in their longitudinal data (20, 21). We hypothesized that latent patterns in HCC values measured longitudinally during early childhood will identify the groups of children at higher risk for altered HPA-axis function. By comparing these groups of children, we sought to identify the maternal and psychosocial factors that may be related to these unique longitudinal HCC profiles. We speculate whether longitudinal measurements of HCC values may identify the children at risk for HPA-axis dysregulation and its longterm consequences, thereby identifying participants for future therapeutic interventions to overcome the pernicious and persistent effects of ELS across the lifespan.

## Materials and Methods

### CANDLE Cohort

Following approval from the Institutional Review Board (IRB) at the University of Tennessee Health Sciences Center, participants or legal guardians gave informed consent for participation in the conditions affecting neurocognitive development and learning in Early childhood (CANDLE) study as well as hair sampling from the CANDLE children during yearly clinic visits. Pregnant women (*N* = 1,503) were enrolled during their second trimester between 2006 and 2011 for a longitudinal birth cohort study. CANDLE study participants were described previously (17–19, 22) with detailed descriptions of the instruments and survey items for characterizing children and their mothers (17–19, 22, 23). Characteristics of the children and mothers who gave consent for hair sampling are listed in Supplementary Table A; the variables measured from clinic visits (CV) centered at ages 1, 2, 3 years are listed in Supplementary Table B. Parents declined hair sampling for children on some annual visits due to cosmetic reasons, e.g., preference for buzz cuts for boys or elaborate hair designs for girls.

### Hair Collection, Processing, and HCC Quantification

Hair samples were collected from the posterior vertex by trimming hair close to the scalp surface. Hair samples (10 mg) were cut to powder consistency and proteins extracted by sequential methanol (52°C for ~15 h) acetone (10 min at room temperature) extractions, repeated once. Supernatants were air evaporated at 4°C; the protein residue reconstituted in 70 mcl of phosphate buffered saline (PBS, pH 7.8) per 10 mg hair, kept at 4°C for immediate use or at −20°C for long-term storage. The Pierce Micro BCATM Protein Reagent Kit was used to determine total protein content and the Alpco Cortisol (Saliva) ELISA assay was used to quantify HCC. An Epoch BioTek plate reader with associated software Gen 5 Biotech (Version 1.11, Winooski, VT, USA) determines total protein and cortisol levels (ng/mg hair) (16). HCC assays had intra-assay and inter-assay coefficients of variation at <4 and <8%, respectively.

### Statistical Methods

The Stanford University IRB approved these data analyses. Data analyses were conducted on CANDLE subjects providing hair samples at 2 or 3 clinic visits (CV) with age at CV1 = 11–18 months; CV2 = 23–30 months; and CV3 = 35–42 months. Longitudinal HCC values were examined visually via scatterplot and log-transformed HCC values (ln-HCC) were partitioned into different trajectories using LCMM (20, 21). The model was framed as a linear mixed effects model for ln-HCC as a function of fixed effects for age in months and age in months squared, coupled with random effects for CANDLE participants (to account for repeated measures from the same subject) and latent classes identified by age in months. The number of latent classes were pre-specified to 2, 3, 4, or 5, and the pre-specified value that minimized the Bayesian Information Criterion (BIC) was selected (Supplementary Table C). Vrieze states that “BIC has α = log(*N*), where *N* is the number of observations contributing to the sum in the likelihood equation. The number of model parameters κ is taken to be a direct indicator of model complexity; the more parameters the more flexible and complex the model” (24).

The same process of fitting LCMM models within subgroups was repeated until consistent ln-HCC patterns remained within each identified group. This approach is commonly recommended in various statistical texts (25–27) and in a reference text: *“Applied Latent Class Analysis”* edited by Hagenaars and McCutcheon (28). These authors state that LCMM analyses may be performed sequentially or simultaneously within the subclasses identified from an initial model. Sequential LCMM modeling procedures within stratified subpopulations are also reported in several previous studies (29–31).

Associations between candidate variables and the LCMM-identified classes were examined *via* univariate and multivariate tests at each clinic visit (Supplementary Tables D–F). Associations between each candidate variable and group membership were tested using Fisher's exact test for categorical variables and Wilcoxon-rank sum for continuous variables. Candidate variables with *p* ≤ 0.1 were entered into logistic regression models using backwards selection to eliminate the variable with the largest *p*-value at each step until all the remaining variables were *p* < 0.1. Variable selection was implemented in the R package “rms” (Regression Modeling Strategies; R package version 5.1-3.1.). All statistical analyses were conducted using R version 3.6.1 (32). Study integrity was assured by completing the STROBE checklist for observational studies (Supplementary Table G) and the GRoLTS checklist for latent trajectory modeling studies (Supplementary Table H).

## Results

Visual inspection of the ln-HCC values plotted by age in months (Supplementary Figure 1) illustrated the complexity of longitudinal ln-HCC trajectories for individual children with varying exposures to contextual stressors over time.

### Latent Classes 1 and 2

**Figure 1:**
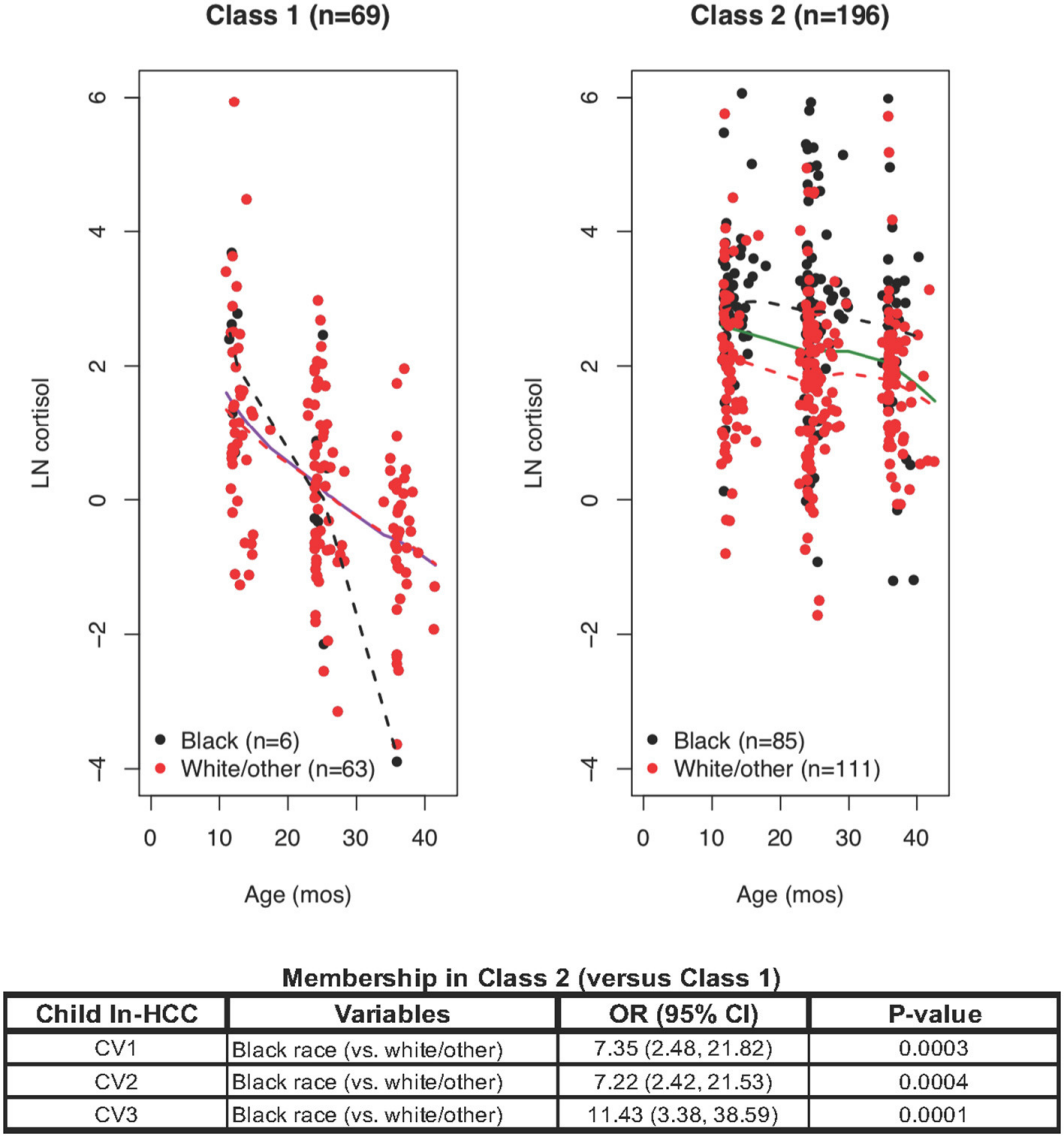
Latent class mixed modeling (LCMM) revealed Class 1 and Class 2: ln-HCC values plotted by age in months, with solid regression lines for all children, dashed regression lines for Blacks (black) and White/other races (red). A logistic regression model revealed that Class 2 membership was determined by Black race at each clinic visit (CV).

The initial LCMM model yielded two latent classes: Class 1 with steadily declining ln-HCC values across age (*n* = 69, 9% Black); and Class 2 with little or no changes in ln-HCC across early childhood (*n* = 196, 43% Black). Class 1 showed the expected trajectory of declining ln-HCC values across early childhood as reported previously (18, 33) (Figure 1). Compared to Class 2, the mothers of **Class 1** children were more likely to be married (81.2 vs. 57.1% at CV1, 82.6 vs. 57.1% at CV2, 81.2 vs. 53.6% at CV3), White/other race (87 vs. 55.6%), older (29.3 ± 4.6 vs. 27.8 ± 5.0 years), they had lower body weights before (70.8 ± 15.5 vs. 76.8 ± 20.5 kg*)* and during pregnancy (77.5 ± 16.1 vs. 84.4 ± 20.1 kg), higher breastfeeding rates at 4 weeks post-partum (69.6 vs 57.7%), higher household incomes ($65,000 or more, 46.4 vs. 27.6%), and private health insurance (76.8 vs. 59.2%).

*Class 1 mothers* also reported fewer traumatic events in the Traumatic Life Events Questionnaire (TLEQ) during pregnancy (2.6 ± 2.2 vs. 3.4 ± 2.4) and at CV3 (2.3 ± 2.4 vs. 3.1 ± 2.5), fewer distress symptoms in the Brief Symptom Inventory (BSI, 50.8 ± 7.3 vs. 53.1 ± 8.1), greater knowledge of infant development (0.8 ± 0.1 vs. 0.7 ± 0.1), lower scores on the Child Abuse Potential Index (CAPI) for rigidity (9.8 ± 11.4 vs. 15.2 ± 13.4) or abusive parenting (58.8 ± 70.1 vs. 73.8 ± 65.6), and fewer indicators of unhappiness (7.2 ± 7.8 vs. 9.3 ± 9.3). Fewer fathers of children in Class 1 than in Class 2 had a history of smoking (15.9 vs. 29.6%).

*Class 1 children* had fewer social-emotional problems (Brief Infant-Toddler Social-Emotional Assessment, BITSEA, problem total score) at CV1 (7.1 ± 4.4 vs. 9.1 ± 5.7) and CV2 (7.9 ± 7.6 vs. 9.5 ± 6.2), lower scores for externalizing behaviors at CV1 (1.8 ± 1.7 vs. 2.4 ± 2.0) and internalizing behaviors at CV2 (1.6 ± 1.9 vs. 2.1 ± 1.6). Variables reaching a significance level of *p* ≤ 0.1 (see Supplementary Table C) were selected for logistic regression modeling.

**Figure 2:**
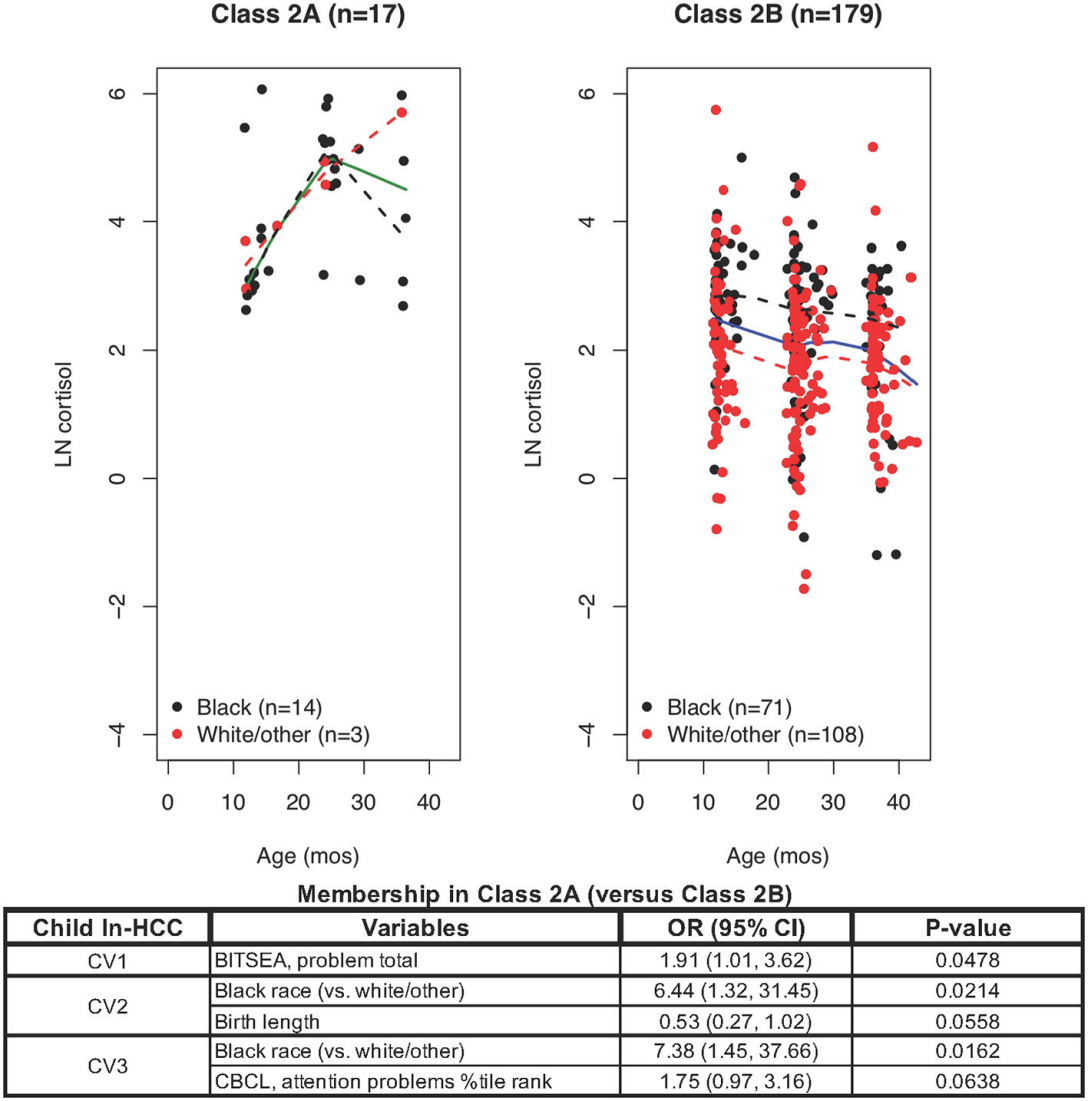
LCMM model only for Class 2 members revealed Class 2A and Class 2B: ln-HCC values plotted by age in months, with solid regression lines for all children, dashed regression lines for Blacks (black) and White/other races (red). Logistic regression showed that Class 2A membership was associated with Black race (CV2, CV3) and greater social-emotional problems (CV1), with trends for shorter body length at birth (CV2) and greater attention problems at CV3.

Logistic regression analyses showed that **Class 2** (vs. Class 1) membership was only dependent on Black race at CV1 (OR = 7.35, *p* = 0.0003), CV2 (OR = 7.22, *p* = 0.0004), and CV3 (OR = 11.43, *p* = 0.0001; Figure 2). Further analyses were warranted given the substantial variability of ln-HCC values in Class 2 and divergence of ln-HCC trajectories between races.

### Latent Classes 2A and 2B

LCMM partitioning of ln-HCC values within Class 2 revealed Class 2A with 17 members (82% Black) showing elevated and increasing ln-HCC values over time (Figure 2) and 179 children in Class 2B (40% Black) with high variability in their ln-HCC trajectories. Compared to Class 2B, Class 2A members had more Black children (82.4 vs. 39.7%), shorter body length (47.9 ± 4.5 cm vs. 50.8 ± 2.5 cm) and need for NICU care at birth (23.5 vs. 5.0%). Class 2A members also showed higher BITSEA scores for social-emotional problems at CV1 and CV2 (14.8 ± 8.2 vs. 8.6 ± 5.1 and 12.1 ± 6.0 vs. 9.3 ± 6.2, respectively), higher internalizing (2.9 ± 1.4 vs. 1.7 ± 1.4) and externalizing (3.9 ± 2.6 vs. 2.2 ± 1.9) behaviors at CV1, as well as more frequent problems with aggression (1.2 ± 0.5 vs. 1.0 ± 0.2) and attention (69.1 ± 16.2 vs. 62.1 ± 13.9) at CV3 as measured in the Child Behavior Checklist (CBCL). Mothers of children in Class 2A vs. 2B were less likely to breastfeed at 4 weeks (29.4 vs. 60.3%), had lower household incomes (>$65,000, 5.9 vs. 29.6%), greater somatization at CV1 (52.0 ± 8.8 vs. 47.8 ± 8.4), and higher abuse potential at CV3 (102.0 ± 80.6 vs. 72.1 ± 70.2).

Logistic regression analyses showed that variables determining **Class 2A** (vs. Class 2B) membership included a shorter body length at birth (OR = 0.53, *p* = 0.0558), social-emotional problems at CV1 (OR = 1.91; *p* = 0.0478), Black race at CV2 and CV3 (OR = 6.44, *p* = 0.0214; OR = 7.38, *p* = 0.0162, respectively), and greater affective problems at CV3 (OR = 1.75, *p* = 0.0638). Further analysis of Class 2B was warranted given the large class size (*n* = 179), observable differences in ln-HCC trajectories by race, and significant variability in the ln-HCC values.

### Latent Classes 2B1 and 2B2

**Figure 3:**
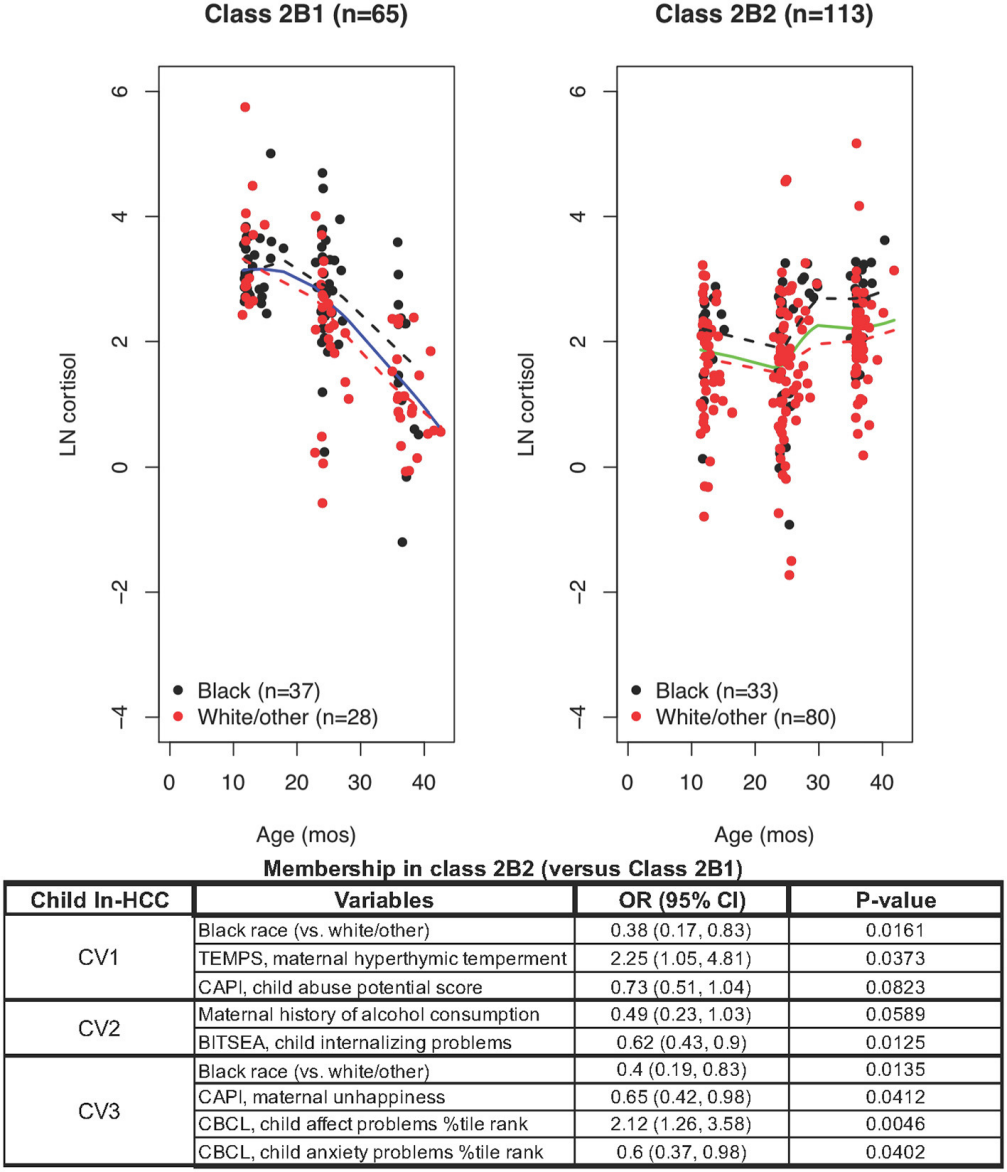
LCMM model exclusively including Class 2B members revealed Class 2B1 and Class 2B2: ln-HCC values plotted by age in months, with solid regression lines for all children, dashed regression lines for Blacks (black) and White/other races (red). Logistic regression revealed that ln-HCC in Class 2B2 was associated with White/other race (CV1, CV3), maternal hyperthymic temperament (CV1) and unhappiness (CV3), the child's internalizing behaviors (CV2), affect and anxiety problems (CV3).

LCMM modeling limited exclusively to Class 2B members revealed Class 2B1 (*n* = 65) with high but declining ln-HCC values and Class 2B2 (*n* = 113) with sustained elevations in ln-HCC values (Figure 3). Compared to Class 2B2, *mothers in* ***Class 2B1*** were less likely to be White/other race (43.1 vs. 69.0%) or have private insurance (50.8 vs. 66.4%); they experienced more ACEs in the TLEQ (0.7 ± 0.9 vs. 0.4 ± 0.8), greater physical aggression during pregnancy on the Conflict Tactics Scale (3.6 ± 4.1 vs. 2.3 ± 3.7), and based on their CAPI scores, showed greater rigidity at CV1 and CV3 (18.2 ± 14.7 vs. 12.7 ± 11.7 and 17.3 ± 15.4 vs. 12.1 ± 11.9, respectively) and abusive parenting styles at every age (CV1: 89.7 ± 80.5 vs. 62.1 ± 50.9; CV2: 85.7 ± 82.2 vs. 65.9 ± 67.1; CV3: 86.6 ± 81.8 vs. 62.7 ± 60.6). The children in Class 2B1 had shorter body length at birth (50.2 ± 2.6 cm vs. 51.2 ± 2.5 cm), higher internalizing behaviors at CV2 (2.5 ± 1.7 vs. 1.8 ± 1.5), and higher anxiety scores at CV3 (60.3 ± 13.6 vs. 57.4 ± 13.7).

A logistic regression model showed that mothers of children in **Class 2B2** were less likely to be Black (CV1, OR = 0.38, *p* = 0.0161; CV3, OR = 0.4, *p* = 0.0135), had a hyperthymic temperament (OR = 2.25, *p* = 0.0373) at CV1, with less unhappiness (CV3, OR = 0.65; *p* = 0.0412) than Class 2B1 mothers. The children of Class 2B2 showed less internalizing behaviors (OR = 0.62, *p* = 0.0125) and more affective problems at CV2 (OR = 2.12, *p* = 0.0046), and less anxiety problems at CV3 (OR = 0.6, *p* = 0.0402).

## Discussion

Karlen et al. found that sequential HCC measurements in children aged 1, 3, 5, and 8 years had significant linear correlations across ages, but they did not examine longitudinal trajectories in individual children or relate these effects to ELS (33). Our cross-sectional analyses also revealed that higher ln-HCC in the preschool children at earlier ages significantly predicted their ln-HCC values at subsequent ages (18). Consequently, we conducted longitudinal analyses to reveal latent patterns in their ln-HCC trajectories and to examine the maternal and child-related factors that may determine membership in these latent classes.

We present the first-ever longitudinal analyses of hair cortisol from a geographically defined and racially diverse urban/suburban population of children aged 1-3 years, identifying latent classes based on their ln-HCC trajectories. We previously reported cross-sectional analysis of ln-HCC in this age group, showing that Black children were exposed to greater adversity (17) leading to chronic stress (18, 19, 22). Here, we extend our studies to explore latent patterns in ln-HCC trajectories from the children with longitudinal data. Membership in latent classes based on ln-HCC trajectories defined groups of children with progressive profiles of altered HPA-axis function, presumably resulting from increasing allostatic load as suggested by maternal and psychosocial factors as well as their physical and behavioral outcomes (14, 34).

The typically developing human HPA-axis matures by 4 years, but it remains malleable before age 4. Therefore, it is important to determine the trajectories indicative of HPA-axis function in earlier developmental windows, when the set-points for long-term HPA-axis regulation are vulnerable to contextual stressors in early life (15, 34, 35), predisposing children to progress from adaptive, to maladaptive, and to toxic stress responses (5). Adaptive stress responses promote mastery and help build a child's resilience, maladaptive stress responses may interrupt these developmental processes, whereas toxic stress responses disable resilience and problem-solving skills, promoting passive submission or self-injurious/risk-taking behaviors (1, 4, 36).

Children assigned to LCMM Class 1 had decreasing ln-HCC across ages 1-3 years (Figure 1), following the overall patterns of HPA-axis function expected in normal healthy children (18). Karlen et al. confirmed a pattern of decreasing HCC during early childhood (33), which was not seen in cross-sectional studies with sparse sampling from the preschool age groups (37). Given their favorable demographic and psychosocial factors, we propose that Class 1 identifies those children with an adaptive profile of HPA-axis function, easily capable of maintaining homeostasis after exposure to contextual stressors. Class 1 children had fewer social-emotional problems in infancy and manifested fewer internalizing or externalizing behaviors than those in Class 2. Other studies also found impaired social-emotional development associated with ELS (38, 39), further linked with the poorer cognitive and emotional outcomes in older children and adolescents, and psychopathology in adults (4, 40).

Subsequent partitioning of Class 2 identified the children with a hyperresponsive HPA-axis in Class 2A compared to 2B, showing high ln-HCC at age 1 year and further increasing values across early childhood rather than the expected decline in typically developing children (Figure 2). Most of these children were Black, had been exposed to maternal psychosocial risk factors, possibly nutrition-related intrauterine growth restriction at birth (41), and were more likely to develop social-emotional problems, internalizing and externalizing behaviors in infancy, as well as problems with aggression and attention in later childhood (38, 39). HPA-axis hyperreactivity follows failure of the cortisol-mediated negative feedback loop that regulates the secretion of CRH and ACTH (42). These children may have higher risks for developing long-term HPA-axis dysregulation, epidemiologically linked with early obesity, metabolic syndrome, adult-type NCDs (1, 11, 43) and psychopathology (4, 40, 44). Another study measuring salivary cortisol levels revealed that “*Race differences in cortisol were not present at 4 months but emerged at 12 months of age, with Black infants having higher cortisol… suggesting that racial discrimination is already “under the skin” by 1 year of age”* (45). We previously reported preliminary evidence for HPA-axis dysregulation in 1-year-old Black infants (19), and now present further evidence for HPA-axis dysfunction from longitudinal HCC trajectories in Class 2A mostly including 1-3 year-old Black children (82.4%), as compared to Class 1 membership mostly including White/other children (91.3%).

Class 2B1 members also showed the expected decline across early childhood, but their HCC values were higher than in Class 1 (Figure 3 vs. Figure 1) (18, 33). This group contained similar numbers of White/other and Black children, perhaps in the compensatory phase of HPA-axis dysregulation with attempted adaptation to contextual stressors (46). Children in Class 2B1 were exposed to maternal and psychosocial risk factors, evidenced intra-uterine growth restriction at birth and, consistent with previous research, showed more internalizing behaviors at age 2 and anxiety at age 3 (40, 47, 48). Class 2B2, including 70% White/other children, showed a flat trajectory across early childhood—somewhat like Class 2A which mainly included Black children. The mothers of Class 2B2 children uniquely showed a hyperthymic temperament, which may lead to overly intrusive or overprotective parenting, associated with HPA-axis dysregulation in another study (49).

**Figure 4:**
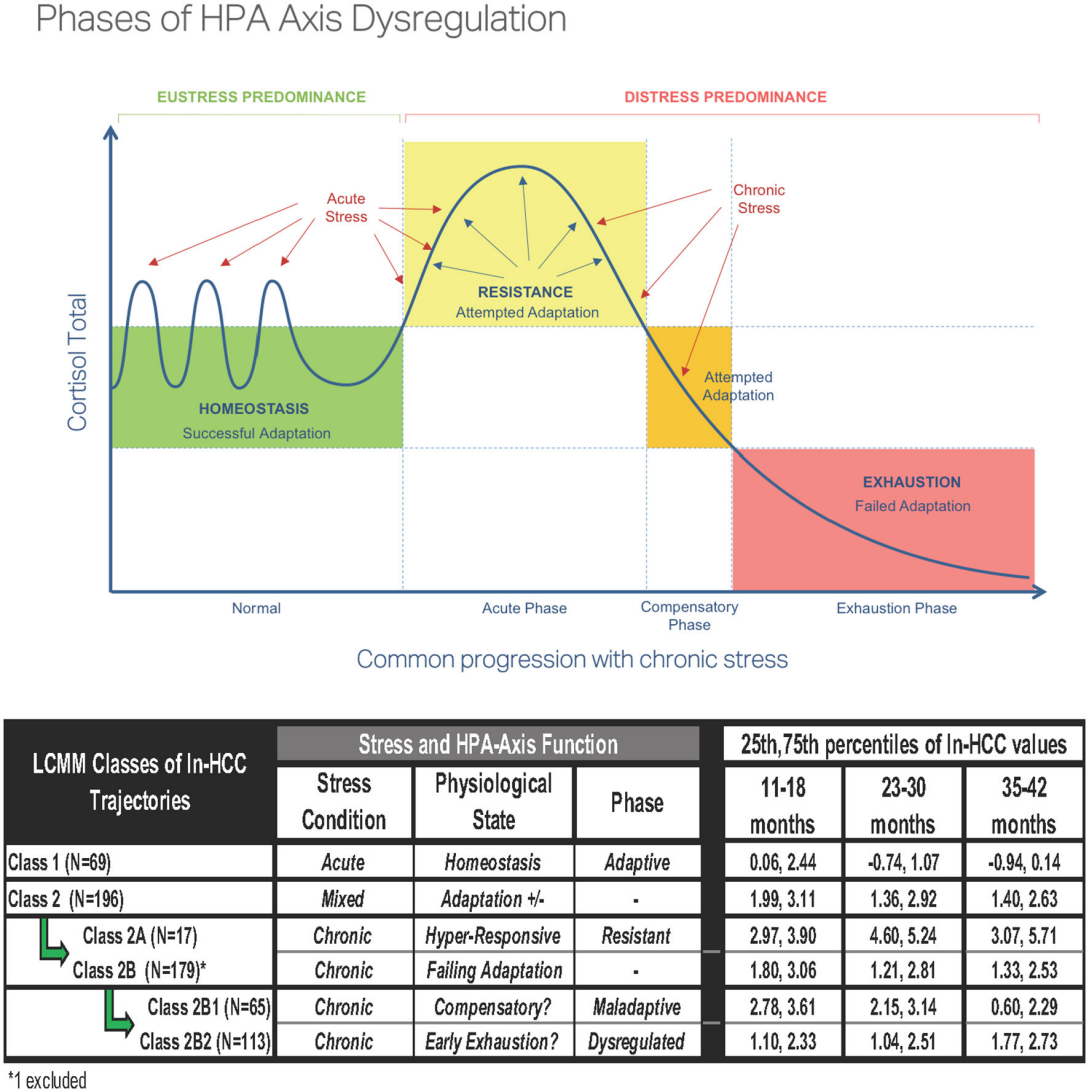
Phases of hypothalamic-pituitary-adrenal (HPA)-axis dysregulation [reproduced with permission from Bella Lindemann (www.bellalindemann.com)] based on animal studies. Eustress predominance under conditions of adaptive stress exposure to normal environmental conditions promotes maturation of the HPA-axis in early life. Distress predominance with worsening exposures to early life adversity in the absence of socially affiliative/nurturing relationships leads to a maladaptive (hyperresponsive) HPA-axis and delayed return to homeostasis. Severe or multiple chronic stressors can disrupt cortisol secretion with the hypocortisolemia associated with HPA-axis dysregulation. Proposed variations in HPA-axis function for the LCMM Classes showing the 25th and 75th percentiles of ln-HCC values in each latent class.

The interplay of health and psychosocial determinants occurs in a bidirectional fashion; therefore, correlates of HCC in toddlers may become prognosticators of later health outcomes. Given the patterns suggestive of HPA dysfunction in Classes 2A, 2B1, and 2B2, we speculate that children experiencing repeated contextual stressors may develop maladaptive HPA-axis responses that were associated with depression or posttraumatic stress disorder (PTSD) (48, 50–53). Lower HCC values were also noted in individuals with PTSD as compared to normal or acute stress groups (54). These findings appear to align with the theoretical phases of HPA-axis dysregulation (Figure 4), in which cumulative allostatic loads related to ELS may progressively impair HPA-axis function, as illustrated in Class 2 and its subclasses. The current criteria for HPA-axis dysregulation are based on plasma and salivary cortisol levels, which are measures of acute stress which can be altered by diurnal cycles, high state reactivity, food intake, pulsatile secretion patterns, superimposed on the robust changes across age, sex, reproductive cycles, and pubertal stages (12, 13, 55, 56). Because prolonged or chronic stressors are more likely precursors of HPA-axis dysfunction than acute stressors, putative criteria for identifying HPA-axis dysregulation should also be developed from HCC, which serves as a summative measure of chronic stress (57). Based on our data, we present a starting point for the HCC ranges in 1-3 year-old children that could be used for developing such criteria (Figure 4).

The results of these analyses must be interpreted in the light of four limitations. First, as in other cohort studies of early stress (19, 58, 59), the generalizability of these results is limited by the differences in demographic characteristics between children with HCC measurements in the CANDLE study and those of other children (17). Second, HCC data from all clinic visits were only present in 45 children, with other children contributing measurements at any combination of two timepoints. This highlights the challenges of implementing longitudinal studies in a cohort of inner-city children and obtaining consent for hair sampling at each of their clinic visits. Though missing data may affect the confidence intervals of the HCC trajectories identified, our use of LCMM modeling was specifically suited for handling missing data in our subjects (21, 25, 26, 28). Third, because these are exploratory analyses to identify the characteristics of LCMM class membership, we did not adjust the *p*-values for multiple testing. Therefore, the explanatory factors identified in our univariate or logistic regression analyses must be confirmed in larger cohort studies. Fourth, backwards selection is one of many variable selection techniques that can be used, each with its own limitations. Whereas elastic net may be superior for minimizing prediction error, our choice of backwards selection was designed to identify those variables that retain significant associations with class membership. Our sensitivity analyses included variable selection using LASSO and elastic net, and found that similar variables were selected consistently across all methods. However, our use of logistic regression modeling with backwards selection allows easier interpretability of the preliminary variables that characterize latent class membership.

We present an objective method for longitudinally measuring chronic stress in a cohort of children aged 1–3 years, identify factors associated with adaptive and altered forms of HPA function, and suggest an approach to interpreting HCC values in this cohort. These results expand on our previous publications on HCC and social adversity in preschool children, clearly demarcating racial and socioeconomic disparities in urban/suburban populations (17, 18). Longitudinal measures of HCC in young children may provide an objective tool for classifying the early developmental trajectories in immature stress pathways and could also be used assess the efficacy of therapeutic interventions. Taken together, our linked approaches for measuring early life adversity and using HCC trajectories to identify associated risk factors in early childhood may ultimately enable monitoring the effects of policy reform developed to address the widening health inequities between racial, socioeconomic, immigrant, and other demographic subgroups.

## Data Availability Statement

The datasets presented in this study can be found in online repositories. The names of the repository/repositories and accession number(s) can be found at: Data for the CANDLE Study (Conditions Affecting Neurocognitive Development and Learning in Early childhood) are available through https://candlestudy.uthsc.edu.

## Ethics Statement

The studies involving human participants were reviewed and approved by Institutional Review Board (IRB) at the University of Tennessee Health Sciences Center. Written informed consent to participate in this study was provided by the participants' legal guardian/next of kin.

## Author Contributions

CR contributed to the study conceptualization and methodology, data curation, analyses and interpretation, drafting the initial manuscript, making critical revisions, and final approval of the submitted manuscript. JR contributed to statistical design and methodology, data curation, analyses and interpretation, drafting the initial manuscript, making critical revisions, and final approval of the submitted manuscript. J-MR and VC contributed to data curation, analyses and interpretation, making critical revisions, and final approval of the submitted manuscript. MR contributed to data curation, analyses and interpretation, drafting the manuscript, making critical revisions, and final approval of the submitted manuscript. KA obtained grant funding and contributed to study conceptualization, statistical design and methodology, data curation, analyses and interpretation, supervision of the team, drafting of the initial manuscript, making critical revisions, and final approval of the submitted manuscript. All authors contributed to the article and approved the submitted version.

## Funding

The current studies received funding from the Maternal and Child Health Research Institute at Stanford University (P.I. KA) and the *Eunice Kennedy Shriver* National Institute for Child Health and Human Development (R01 HD099296, P.I. KA). Study sponsors had no role in the design and conduct of the study; the collection, management, analysis, or interpretation of the data; the preparation, review, approval, or decision to publish this manuscript.

## Conflict of Interest

The authors declare that the research was conducted in the absence of any commercial or financial relationships that could be construed as a potential conflict of interest.

## Publisher's Note

All claims expressed in this article are solely those of the authors and do not necessarily represent those of their affiliated organizations, or those of the publisher, the editors and the reviewers. Any product that may be evaluated in this article, or claim that may be made by its manufacturer, is not guaranteed or endorsed by the publisher.
