# Supplementary material for: Longitudinal Trajectories of Hair Cortisol: Hypothalamic-Pituitary-Adrenal Axis Dysfunction in Early Childhood
Source: Front Pediatr. 2021 Oct 11;9:740343. doi: 10.3389/fped.2021.740343 (PMC8544285; doi:10.3389/fped.2021.740343)
Supplement: Supplementary file 1 [file Data_Sheet_1.PDF]

CODE FOR SUPPLEMENTARY TABLES B-F: Significance level for reporting variables included in Supplementary Tables (Fisher's exact test for categorical variables; Wilcoxon rank-sum test for continuous variables; p-value $\leq$ 0.1**).**

Abbreviations**:** Enrollment clinic visit at 16-26 weeks pregnant (M1); 3rd trimester clinic visit at 27-42 weeks pregnant (M2); birth newborn (M3); home visit at one month post-partum (HV1); child annual clinic visits (CV)- CV1 (11-18 months), CV2 (23-30 months), and CV3 (35-42 months).

Demographic Questionnaire (DEM); Temperament Evaluation (TEMPS), Brief Symptom Index (BSI); Edinburgh Post-Natal Depression Scale (EPDS); Rosenberg Self-Esteem (RSE); Conflict Tactics Scale (CTS); Knowledge of Infant Development Inventory (KIDI); Child Abuse Potential Inventory (CAPI); Traumatic Life Events Questionnaire (TLEQ); Labor and Delivery Summary/Complications (LD), Neonatal Summary Form (NSF); Family History Inventory (FHI); BAYLEY Scales of Infant Development III (BAY); Brief Infant-Toddler Social and Emotional Assessment (BITSEA); Demographic Survey (Dem); NEO Personality Inventory (NEO).
