# Supplementary material for: Longitudinal Trajectories of Hair Cortisol: Hypothalamic-Pituitary-Adrenal Axis Dysfunction in Early Childhood
Source: Front Pediatr. 2021 Oct 11;9:740343. doi: 10.3389/fped.2021.740343 (PMC8544285; doi:10.3389/fped.2021.740343)
Supplement: Supplementary file 2 [file Data_Sheet_2.PDF]

Supplementary Table A: Demographics of the Research Subjects

**Clinic Visit:**<br>Number of subjects:	**CV1**<br>n=170	**CV2**<br>n=238	**CV3**<br>n=167
**Age (years)**	1.1 (±0.1)	2.1 (±0.1)	3.1 (±0.1)
**Sex**			
Male	56 (32.9%)	84 (35.3%)	56 (33.5%)
Female	114 (67.1%)	154 (64.7%)	111 (66.5%)
**Race**			
White/other	105 (61.8%)	158 (66.4%)	123 (73.7%)
Black	65 (38.2%)	80 (33.6%)	44 (26.3%)
**Birthweight (grams)**	3260.9 (±515.3)	3351.7 (±502.2)	3375.2 (±466.2)
**Gestational age (weeks)**	38.9 (±1.4)	39.0 (±1.3)	38.9 (±1.5)
**Mother's age (years)**	27.9 (±5.1)	28.2 (±4.9)	28.6 (±4.6)
**Mother's education**			
< High School	4 (2.4%)	5 (2.1%)	4 (2.4%)
High School/GED	66 (38.8%)	81 (34.0%)	41 (24.6%)
Technical School	13 (7.6%)	16 (6.7%)	15 (9.0%)
College Degree	54 (31.8%)	82 (34.5%)	56 (33.5%)
Grad/Professional	33 (19.4%)	54 (22.7%)	51 (30.5%)
**Marital status**			
Married/partner	117 (68.8%)	174 (73.1%)	131 (78.4%)
Single/other	53 (31.2%)	63 (26.5%)	36 (21.6%)
Missing		1 (0.4%)	
**Income**			
>65K	52 (30.6%)	87 (36.6%)	78 (46.7%)
25-65K	56 (32.9%)	76 (31.9%)	50 (29.9%)
<25K	58 (34.1%)	67 (28.2%)	35 (21.0%)
Missing	4 (2.4%)	8 (3.4%)	4 (2.4%)
**Insurance**			
Private	107 (62.9%)	155 (65.1%)	117 (70.1%)
Public/other	63 (37.1%)	83 (34.9%)	50 (29.9%)

Note: Hair Cortisol Concentrations (HCC) were measured in 265 children at annual Clinic Visits (CV) centered at 1, 2, and 3 years (HCC values N=575). Age ranges at: CV1 (11-18 months), CV2 (23-30 months), and CV3 (35-42 months). Number of children with HCC data at: CV1, CV2, CV3 (n=45); CV1 and CV2 (n=98); CV1 and CV3 (n=27); CV2 and CV3 (n=95).
