# Supplementary material for: Longitudinal Trajectories of Hair Cortisol: Hypothalamic-Pituitary-Adrenal Axis Dysfunction in Early Childhood
Source: Front Pediatr. 2021 Oct 11;9:740343. doi: 10.3389/fped.2021.740343 (PMC8544285; doi:10.3389/fped.2021.740343)
Supplement: Supplementary file 3 [file Data_Sheet_3.PDF]

Supplementary Table B: Family, maternal, and child variables were used to assess their predictive capacity for longitudinal trajectories of hair cortisol concentration. The percent missing data in the 265 children profiled for the longitudinal dataset are reported for each annual clinic visit (CV).

	Percent missing		
	**CV1**	**CV2**	**CV3**
Family Variables	**65 variables**	**61 variables**	**74 variables**
FHI_ Father has history of Alcohol abuse problem or disorder	0	0	0
FHI_ Father has history of Drug abuse problem or disorder	0	0	0
FHI_ Father has history of Smoking	0	0	0
FHI_ Mother has history of Alcohol abuse problem or disorder	0	0	X
FHI_ Mother has history of Drug abuse problem or disorder	0	0	0
FHI_ Mother has history of Smoking	0	0	0
FHI_ Brother/sister has history of Alcohol abuse problem or disorder	0	0	X
FHI_ Brother/sister has history of Drug abuse problem or disorder	0	0	X
FHI_ Brother/sister has history of Smoking	0	0	X
DEM_ Race	0	0	0
DEM_ Current married/partner status	CV1, 3	CV2, 0.8	CV3, 3.4
DEM_ Estimated total annual household income	CV1, 4.5	CV2, 3.4	CV3, 5.7
DEM_ Health insurance coverage (private/other)	CV1, 3.8	CV2, 0.4	CV3, 3.4
Maternal	***CV1***	***CV2***	***CV3***
M1, Alcohol use during pregnancy	0	0	0
M1, Body mass index	0	0	0
M1, DEM_ Age	0	0	0
M1, Diabetes (ever)	0.4	0.4	0.4
M1, Diabetes current pregnancy	0	0	X
M1, Height (cm)	0	0	0
M1, Pre-pregnancy weight (kg)	0	0	0
M1, Number of pregnancies delivered pre-term (< or = 37 weeks)	0	X	X
M1, Pregnancy weight (kg)	0.4	0.4	0.4
TEMPS_ Cyclothymic temperament	M1, 0	CV2, 0.8	CV2, 0.8
TEMPS_ Dysthymic temperament	M1, 0	CV2, 0.8	CV2, 0.8
TEMPS_ Hyperthymic temperament	M1, 0	CV2, 0.8	CV2, 0.8
TEMPS_ Irritable temperament	M1, 0	CV2, 0.8	CV2, 0.8
M1, Tobacco use during pregnancy	0	0	0
M1, Total number of pregnancies (including current pregnancy, miscarriages, abortions, and stillbirths)	0	0	0
M1, Total pregnancy sum	0	0	0
BSI_T-score for Global Severity Index	M2, 2.6	CV2, 2.6	X
BSI_T-score for Positive Symptom Distress Index	M2, 2.6	CV2, 2.6	X

BSI_T-score for Positive Symptom Total	M2, 2.6	CV2, 2.6	X
BSI_ T-score for Somatization scale	X	CV2, 4.2	X
BSI_ Sum of all scale sums and additional items response sum	CV1, 4.2	CV2, 0.4	CV3, 4.5
CTS_ Frequency of Injury Score Self	2.6	X	X
CTS_ Frequency of Physical Aggression Score Partner	M2, 2.6	CV2, 0.8	CV2, 0.8
CTS_ Frequency of Physical Aggression Score Self	M2, 2.6	CV2, 0.8	CV2, 0.8
CTS_ Frequency of Psychological Aggression Score Partner	M2, 2.6	CV2, 0.8	CV2, 0.8
CTS_ Frequency of Psychological Aggression Score Self	M2, 2.6	CV2, 0.8	CV2, 0.8
M2, KIDI_ Total overall score of questions answered correctly	M2, 2.6	2.6	2.6
RSE_ Score	M2, 2.6	CV2, 0.8	CV2, 0.8
TLEQ_ Count of Adverse Child Events	M2, 2.6	2.6	CV3, 4.5
TLEQ_ Count of Adverse Events (Adult)	M2, 2.6	2.6	CV3, 4.5
M3, LD_ Psychological stress reported since last visit	2.3	2.3	2.3
HV1, History of alcohol consumption	7.5	7.5	7.5
HV1, Smoking cigarettes now	7.5	7.5	7.5
HV1, Pregnant or breastfeeding	7.5	7.5	7.5
HV1, Self-report of post-partum health status (likert scale?)	7.5	7.5	7.5
CAPI_ Abuse Scale Total Score	CV1, 3.8	CV2, 0.4	CV3, 4.2
CAPI_ Distress Scale Total Score	CV1, 3.8	CV2, 0.4	CV3, 4.2
CAPI_ Rigidity Scale Total Score	CV1, 3.8	CV2, 0.4	CV3, 4.2
CAPI_ Unhappiness Scale Total Score	CV1, 3.8	CV2, 0.4	CV3, 4.2
CV1, EPDS_ Total score	3	3	3
NEO_ Agreeableness Scale T-score	X	X	CV3, 4.9
NEO_ Conscientiousness scale T-score	X	X	CV3, 4.9
NEO_ Extraversion Scale T-score	X	X	CV3, 4.9
NEO_ Neuroticism scale T-score	X	X	CV3, 4.9
NEO_ Openness scale T-score	X	X	CV3 4.9
<u>Child</u>	***<u>CV1</u>***	***<u>CV2</u>***	***<u>CV3</u>***
M3, NSF_ Birth length	1.5	1.5	1.5
M3, NSF_ Birth weight (g)	0	0	0
M3, NSF_ Child sex	0	0	0
M3, NSF_ Gestational age at birth (weeks)-limit to 20-45	0	0	0
M3, NSF_ Highest level of care required	0	0	0
BAY_ Cognitve Risk Category	CV1, 3	X	X
BAY_ Developmental Disability	CV1, 3	CV2, 0.4	CV3, 3.8
BAY_ Expressive Communication Risk Category	CV1, 3	X	X
BAY_ Receptive Communication Risk Category	CV1, 3	X	X
BITSEA_ Competency Total	CV1, 3.4	CV2, 0.4	CV3, 0.4
BITSEA_ Externalizing subscale, subcomponent of Problem scale	CV1, 3.4	CV2, 0.4	X
BITSEA_ Internalizing subscale, subcomponent of Problem scale	CV1, 3.4	CV2, 0.4	X
BITSEA_ Problem Total	CV1, 3.4	CV2, 0.4	CV3, 0.4
CBCL_ Affective Problems %tile Rank	X	X	CV3, 4.5
CBCL_ Affective Problems Range	X	X	CV3, 4.5

CBCL_ Aggressive Behavior %tile Rank	X	X	CV3, 4.5
CBCL_ Aggressive Behavior Range	X	X	CV3, 4.5
CBCL_ Anxiety Problems %tile Rank	X	X	CV3, 4.5
CBCL_ Anxiety Problems Range	X	X	CV3, 4.5
CBCL_ Anxious/Depressed %tile Rank	X	X	CV3, 4.5
CBCL_ Attention Deficit/Hyperactivity Problems %tile Rank	X	X	CV3, 4.5
CBCL_ Attention Problems %tile Rank	X	X	CV3, 4.5
CBCL_ Attention Problems Range	X	X	CV3, 4.5
CBCL_ Emotionally Reactive %tile Rank	X	X	CV3, 4.5
CBCL_ External Range	X	X	CV3, 4.5
CBCL_ Externalizing %tile Rank	X	X	CV3, 4.5
CBCL_ Internal Range	X	X	CV3, 4.5
CBCL_ Internalizing %tile Rank	X	X	CV3, 4.5
CBCL_ Oppositional Defiant Problems %tile Rank	X	X	CV3, 4.5
CBCL_ Pervasive Developmental Problems %tile Rank	X	X	CV3, 4.5
CBCL_ Sleep Problems %tile Rank	X	X	CV3, 4.5
CBCL_ Somatic Complaints %tile Rank	X	X	CV3, 4.5
CBCL_ Total Problems %tile Rank	X	X	CV3, 4.5
CBCL_ Withdrawn %tile Rank	X	X	CV3, 4.5
