# Supplementary material for: Longitudinal Trajectories of Hair Cortisol: Hypothalamic-Pituitary-Adrenal Axis Dysfunction in Early Childhood
Source: Front Pediatr. 2021 Oct 11;9:740343. doi: 10.3389/fped.2021.740343 (PMC8544285; doi:10.3389/fped.2021.740343)
Supplement: Supplementary file 4 [file Data_Sheet_4.PDF]

Supplementary Table C: Fit statistics (BIC value) for latent class mixture models

	2 classes	3 classes	4 classes	5 classes
**Split 1**	2167.598	2175.086	2182.95	2198.936
**Split 2**	1413.114	1428.948	1444.782	1460.617
**Split 3**	1194.67	1210.216	1225.761	1241.203
