# Supplementary material for: Longitudinal Trajectories of Hair Cortisol: Hypothalamic-Pituitary-Adrenal Axis Dysfunction in Early Childhood
Source: Front Pediatr. 2021 Oct 11;9:740343. doi: 10.3389/fped.2021.740343 (PMC8544285; doi:10.3389/fped.2021.740343)
Supplement: Supplementary file 5 [file Data_Sheet_5.PDF]

Supplementary Table D:

Logistic regression model with p-values for Class 1 vs. Class 2 of longitudinal variables by clinic visit (CV) and category (family, maternal, child).

CV1		Class 2 n=196	Class 1 n=69	P-value
Family				
**M1, DEM_ Race**				
	Black	84 (42.9%)	6 (8.7%)	**<0.0001**
	White	109 (55.6%)	60 (87.0%)	
	Asian	2 (1.0%)	3 (4.3%)	
	Other	1 (0.5%)	0 (0.0%)	
**CV1, DEM_ Estimated total annual household income**				
	<25K	67 (34.2%)	13 (18.8%)	**0.008**
	25-65K	67 (34.2%)	20 (29.0%)	
	>65K	54 (27.6%)	32 (46.4%)	
	Missing	8 (4.1%)	4 (5.8%)	
**CV1, DEM_ Current marital status**				
	Never married	56 (28.6%)	8 (11.6%)	**0.003**
	Divorced	2 (1.0%)	0 (0.0%)	
	Separated	5 (2.6%)	0 (0.0%)	
	Living with partner	16 (8.2%)	2 (2.9%)	
	Married	112 (57.1%)	56 (81.2%)	
	Missing	5 (2.6%)	3 (4.3%)	
**CV1, Health insurance coverage**				
	Public	74 (37.8%)	12 (17.4%)	**0.002**
	Private	116 (59.2%)	53 (76.8%)	
	Missing	6 (3.1%)	4 (5.8%)	
**FHI_ Father has history of Smoking**				
	No	138 (70.4%)	58 (84.1%)	**0.026**
	Yes	58 (29.6%)	11 (15.9%)	
Maternal				
**M2, KIDI_ Total overall score of questions answered correctly**		0.7 (±0.1)	0.8 (±0.1)	**0.0004**
	Missing	6 (3.1%)	1 (1.4%)	
**CV1, CAPI_ Rigidity Scale Total Score**		15.2 (±13.4)	9.8 (±11.4)	**0.002**

Missing	7 (3.6%)	3 (4.3%)	
**CV1, CAPI_ Abuse Scale Total Score**	73.8 (±65.6)	58.8 (±70.1)	**0.005**
Missing	7 (3.6%)	3 (4.3%)	
**M2, TLEQ_ Count of Adverse Events (Adult)**	3.4 (±2.4)	2.6 (±2.2)	**0.011**
Missing	6 (3.1%)	1 (1.4%)	
**M1, Pregnancy weight (kg)**	84.4 (±20.1)	77.5 (±16.1)	**0.019**
Missing	0 (0%)	1 (1.4%)	

CV2	**Class 2** n=196	**Class 1** n=69	**P-value**
Family			
**M1, DEM_ Race**			
Black	84 (42.9%)	6 (8.7%)	**<0.0001**
White	109 (55.6%)	60 (87.0%)	
Asian	2 (1.0%)	3 (4.3%)	
Other	1 (0.5%)	0 (0.0%)	
**CV2, DEM_ Estimated total annual household income**			
<25K	66 (33.7%)	11 (15.9%)	**0.001**
25-65K	64 (32.7%)	20 (29.0%)	
>65K	58 (29.6%)	37 (53.6%)	
Missing	8 (4.1%)	1 (1.4%)	
**CV2, DEM_ Current marital status**			
Never married	54 (27.6%)	6 (8.7%)	**0.001**
Divorced	5 (2.6%)	0 (0.0%)	
Separated	6 (3.1%)	0 (0.0%)	
Living with partner	18 (9.2%)	5 (7.2%)	
Married	112 (57.1%)	57 (82.6%)	
Missing	1 (0.5%)	1 (1.4%)	
**CV2, Health insurance coverage**			
Public	80 (40.8%)	14 (20.3%)	**0.002**
Private	115 (58.7%)	55 (79.7%)	
Missing	1 (0.5%)	0 (0.0%)	
**FHI_ Father has history of Smoking**			
No	138 (70.4%)	58 (84.1%)	**0.026**
Yes	58 (29.6%)	11 (15.9%)	
Maternal			

**M2, KIDI_ Total overall score of questions answered correctly**	0.7 (±0.1)	0.8 (±0.1)	**0.0004**
Missing	6 (3.1%)	1 (1.4%)	
**CV2, CAPI_ Rigidity Scale Total Score**	14.8 (±14.8)	9.0 (±11.1)	**0.006**
Missing	1 (0.5%)	0 (0%)	
**CV2, CAPI_ Abuse Scale Total Score**	73.7 (±72.3)	55.1 (±71.2)	**0.007**
Missing	1 (0.5%)	0 (0%)	
**M2, TLEQ_ Count of Adverse Events (Adult)**	3.4 (±2.4)	2.6 (±2.2)	**0.011**
Missing	6 (3.1%)	1 (1.4%)	
**M1, Pregnancy weight (kg)**	84.4 (±20.1)	77.5 (±16.1)	**0.019**
Missing	0 (0%)	1 (1.4%)	
**M2, BSI_T-score for Positive Symptom Distress Index**	53.1 (±8.1)	50.8 (±7.3)	**0.028**
Missing	6 (3.1%)	1 (1.4%)	
**M1, DEM_ Age**	27.8 (±5.0)	29.3 (±4.6)	**0.036**
**HV1, Pregnant or breastfeeding**			
No	69 (35.2%)	15 (21.7%)	**0.046**
Yes	113 (57.7%)	48 (69.6%)	
Missing.	14 (7.1%)	6 (8.7%)	
**M1, Pre-pregnancy weight (kg)**	76.8 (±20.5)	70.8 (±15.5)	**0.061**
**CV2, CAPI_ Unhappiness score**	9.3 (±9.3)	7.2 (±7.8)	**0.037**
Missing	1 (0.5%)	0 (0%)	
Child			
**CV2, BITSEA_ Internalizing subscale, subcomponent of Problem scale**	2.1 (±1.6)	1.6 (±1.9)	**0.001**
Missing	1 (0.5%)	0 (0%)	
**CV2, BITSEA_ Problem Total**	9.5 (±6.2)	7.9 (±7.6)	**0.004**
Missing	1 (0.5%)	0 (0%)	
**CV2, BAY_ Developmental Disability**			
No	175 (89.3%)	55 (79.7%)	**0.047**
Yes	19 (9.7%)	14 (20.3%)	
Missing	1 (0.5%)	0 (0.0%)	

CV3	Class 2 n=196	Class 1 n=69	P-value
Family			

**M1, DEM_ Race**			
Black	84 (42.9%)	6 (8.7%)	**<0.0001**
White	109 (55.6%)	60 (87.0%)	
Asian	2 (1.0%)	3 (4.3%)	
Other	1 (0.5%)	0 (0.0%)	
**CV3, DEM_ Estimated total annual household income**			
<25K	63 (32.1%)	7 (10.1%)	**<0.0001**
25-65K	60 (30.6%)	16 (23.2%)	
>65K	62 (31.6%)	42 (60.9%)	
Missing	11 (5.6%)	4 (5.8%)	
**CV3, DEM_ Current marital status**			
Never married	53 (27.0%)	7 (10.1%)	**0.001**
Divorced	8 (4.1%)	0 (0.0%)	
Separated	5 (2.6%)	1 (1.4%)	
Living with partner	18 (9.2%)	3 (4.3%)	
Married	105 (53.6%)	56 (81.2%)	
Missing	7 (3.6%)	2 (2.9%)	
**CV3_ Health insurance coverage**			
Public	80 (40.8%)	12 (17.4%)	**0.0003**
Private	109 (55.6%)	55 (79.7%)	
Missing	7 (3.6%)	2 (2.9%)	
**FHI_ Father has history of Smoking**			
No	138 (70.4%)	58 (84.1%)	**0.026**
Yes	58 (29.6%)	11 (15.9%)	
Maternal			
**M2, KIDI_ Total overall score of questions answered correctly**	0.7 (±0.1)	0.8 (±0.1)	**0.0004**
Missing	6 (3.1%)	1 (1.4%)	
**CV3, CAPI_ Rigidity Scale Total Score**	14.7 (±14.0)	9.5 (±11.5)	**0.004**
Missing	8 (4.1%)	3 (4.3%)	
**CV3, CAPI_ Abuse Scale Total Score**	74.8 (±71.5)	51.8 (±64.8)	**0.0008**
Missing	8 (4.1%)	3 (4.3%)	
**CV3, CAPI_ Distress Scale Total Score**	36.8 (±50.4)	23.7 (±42.0)	**0.039**
Missing	8 (4.1%)	3 (4.3%)	
**CV3, TLEQ_ Count of Adverse Events (Adult)**	3.1 (±2.5)	2.3 (±2.4)	**0.006**

Missing	9 (4.6%)	3 (4.3%)	
**CV3, TLEQ_ Count of Adverse Events (Child)**	0.4 (±0.8)	0.3 (±0.8)	**0.077**
Missing	9 (4.6%)	3 (4.3%)	
**M1, Pregnancy weight (kg)**	84.4 (±20.1)	77.5 (±16.1)	**0.019**
Missing	0 (0%)	1 (1.4%)	
**M1, DEM_ Age**	27.8 (±5.0)	29.3 (±4.6)	**0.036**
**HV1, Pregnant or breastfeeding**			**0.046**
No	69 (35.2%)	15 (21.7%)	
Yes	113 (57.7%)	48 (69.6%)	
Missing	14 (7.1%)	6 (8.7%)	
**M1_ Pre-pregnancy weight (kg)**	76.8 (±20.5)	70.8 (±15.5)	**0.061**
**CV3, CAPI_Unhappiness score**	9.3 (±9.3)	7.2 (±7.8)	**0.037**
Missing	1 (0.5%)	0 (0%)	
**CV3, NEO_ Extraversion Scale T-score**	52.7 (±10.2)	55.3 (±10.5)	**0.083**
Missing	10 (5.1%)	3 (4.3%)	
Child			
**CV2, BITSEA_ Problem Total**	9.5 (±6.2)	7.9 (±7.6)	**0.004**
Missing	1 (0.5%)	0 (0%)	
