# Supplementary material for: Longitudinal Trajectories of Hair Cortisol: Hypothalamic-Pituitary-Adrenal Axis Dysfunction in Early Childhood
Source: Front Pediatr. 2021 Oct 11;9:740343. doi: 10.3389/fped.2021.740343 (PMC8544285; doi:10.3389/fped.2021.740343)
Supplement: Supplementary file 6 [file Data_Sheet_6.PDF]

Supplementary Table E:

Logistic regression model with p-values for Class 2A vs. Class 2B of longitudinal variables by clinic visit (CV) and category (family, maternal, child).

CV1	Class 2A n=17	Class 2B n=179	P-value
Family			
**M1, DEM_ Race**			
Black	14 (82.4%)	70 (39.1%)	**0.005**
White	3 (17.6%)	106 (59.2%)	
Asian	0 (0.0%)	2 (1.1%)	
Other	0 (0.0%)	1 (0.6%)	
**CV1, DEM_ Estimated total annual household income**			
<25K	8 (47.1%)	59 (33.0%)	**0.088**
25-65K	6 (35.3%)	61 (34.1%)	
>65K	1 (5.9%)	53 (29.6%)	
Missing	2 (11.8%)	6 (3.4%)	
Maternal			
**HV1, Pregnant or breastfeeding**			
No	9 (52.9%)	60 (33.5%)	**0.045**
Yes	5 (29.4%)	108 (60.3%)	
Missing	3 (17.6%)	11 (6.1%)	
**M1, TEMPS_ Cyclothymic temperament**			
No	16 (94.1%)	179 (100.0%)	**0.087**
Yes	1 (5.9%)	0 (0.0%)	
**M2, KIDI_ Total overall score of questions answered correctly**	0.7 (±0.1)	0.7 (±0.1)	**0.096**
Missing	1 (5.9%)	5 (2.8%)	
Child			
**CV1, BITSEA_ Problem Total**	14.8 (±8.2)	8.6 (±5.1)	**0.0009**
Missing	1 (5.9%)	5 (2.8%)	
**CV1, BITSEA_ Internalizing subscale, subcomponent of Problem scale**	2.9 (±1.4)	1.7 (±1.4)	**0.003**
Missing	1 (5.9%)	5 (2.8%)	
**CV1, BITSEA_ Externalizing subscale, subcomponent of Problem scale**	3.9 (±2.6)	2.2 (±1.9)	**0.005**
Missing	1 (5.9%)	5 (2.8%)	
**M3, NSF_ Birth length**	47.9 (±4.5)	50.8 (±2.5)	**0.006**
Missing	0 (0%)	2 (1.1%)	
**M3, NSF_ Highest level of care required**			

Well baby nursery/routine care	13 (76.5%)	170 (95.0%)	**0.017**
NICU/intermediate nursery	4 (23.5%)	9 (5.0%)	
**M3, NSF_ Birth weight (gm)**	2956.5 (±734.2)	3340.9 (±476.0)	0.068
**HV1, FFQ_PREGNANT**			
1	9 (52.9%)	60 (33.5%)	**0.045**
2	5 (29.4%)	108 (60.3%)	
Missing	3 (17.6%)	11 (6.1%)	
**M3, NSF_ Birth weight (g)**	2956.5 (±734.2)	3340.9 (±476.0)	0.068

**CV2**	**Class 2A** n=17	**Class 2B** n=179	**P-value**
Family			
**M1, DEM_ Race**			
Black	14 (82.4%)	70 (39.1%)	**0.005**
White	3 (17.6%)	106 (59.2%)	
Asian	0 (0.0%)	2 (1.1%)	
Other	0 (0.0%)	1 (0.6%)	
Mother			
**CV1, BSI_ T-score for Somatization scale**	52.0 (±8.8)	47.8 (±8.4)	**0.044**
Missing	1 (5.9%)	6 (3.4%)	
**HV1, Pregnant or breastfeeding**			
No	9 (52.9%)	60 (33.5%)	**0.045**
Yes	5 (29.4%)	108 (60.3%)	
Missing	3 (17.6%)	11 (6.1%)	
**M2, KIDI_ Total overall score of questions answered correctly**	0.7 (±0.1)	0.7 (±0.1)	0.096
Missing	1 (5.9%)	5 (2.8%)	
Child			
**M3, NSF_ Birth length (cm)**	47.9 (±4.5)	50.8 (±2.5)	**0.006**
Missing	0 (0%)	2 (1.1%)	
**M3, NSF_ Highest level of care required**			
Well baby nursery/routine care	13 (76.5%)	170 (95.0%)	**0.017**
NICU/intermediate nursery	4 (23.5%)	9 (5.0%)	
**CV2, BITSEA_ Problem Total**	12.1 (±6.0)	9.3 (±6.2)	**0.028**
Missing	0 (0%)	1 (0.6%)	
**M3, NSF_ Birth weight (gm)**	2956.5 (±734.2)	3340.9 (±476.0)	0.068

**CV3**	**Class 2A**	**Class 2B**	**P-value**

	n=17	n=179	
Family			
**M1, DEM_ Race**			
Black	14 (82.4%)	70 (39.1%)	**0.005**
White	3 (17.6%)	106 (59.2%)	
Asian	0 (0.0%)	2 (1.1%)	
Other	0 (0.0%)	1 (0.6%)	
Mother			
**CV3, CAPI_ Abuse Scale Total Score**	102.0 (±80.6)	72.1 (±70.2)	**0.038**
Missing	0 (0%)	8 (4.5%)	
**HV1, Pregnant or breastfeeding**			
No	9 (52.9%)	60 (33.5%)	**0.045**
Yes	5 (29.4%)	108 (60.3%)	
Missing	3 (17.6%)	11 (6.1%)	
**CV3, CAPI_ Distress Scale Total Score**	50.5 (±57.0)	35.5 (±49.7)	**0.091**
Missing	0 (0%)	8 (4.5%)	
**CV3, CAPI_ Rigidity Scale Total Score**	21.5 (±17.8)	14.0 (±13.5)	**0.092**
Missing	0 (0%)	8 (4.5%)	
**M2, KIDI_ Total overall score of questions answered correctly**	0.7 (±0.1)	0.7 (±0.1)	**0.096**
Missing	1 (5.9%)	5 (2.8%)	
Child			
**M3, NSF_ Birth length (cm)**	47.9 (±4.5)	50.8 (±2.5)	**0.006**
Missing	0 (0%)	2 (1.1%)	
**CV2, BITSEA_ Problem Total**	12.1 (±6.0)	9.3 (±6.2)	**0.028**
Missing	0 (0%)	1 (0.6%)	
**CV3, CBCL_ Aggressive Behavior Range**	1.2 (±0.5)	1.0 (±0.2)	**0.036**
Missing	0 (0%)	9 (5.0%)	
**CV3, CBCL_ Attention Problems %tile Rank**	69.1 (±16.2)	62.1 (±13.9)	**0.045**
Missing	0 (0%)	9 (5.0%)	
**M3, NSF_ Birth weight (gm)**	2956.5 (±734.2)	3340.9 (±476.0)	**0.068**
**CV3, CBCL_ Affective Problems %tile Rank**	67.0 (±16.1)	59.4 (±12.0)	**0.078**
Missing	0 (0%)	9 (5.0%)	
**CV3, CBCL_ Attention Deficit / Hyperactivity Problems %tile Rank**	65.4 (±15.5)	61.0 (±13.8)	**0.084**
Missing	0 (0%)	9 (5.0%)	
