# Supplementary material for: Longitudinal Trajectories of Hair Cortisol: Hypothalamic-Pituitary-Adrenal Axis Dysfunction in Early Childhood
Source: Front Pediatr. 2021 Oct 11;9:740343. doi: 10.3389/fped.2021.740343 (PMC8544285; doi:10.3389/fped.2021.740343)
Supplement: Supplementary file 7 [file Data_Sheet_7.PDF]

Supplementary Table F:

Logistic regression model with p-values for Class 2B1 vs. Class 2B2 of longitudinal variables by clinic visit (CV) and category (family, maternal, child).

CV1		Class 2B2 n=113	Class 2B1 n=65	P-value
Family				
**M1, DEM_ Race**				
	Black	33 (29.2%)	36 (55.4%)	**0.0005**
	White	78 (69.0%)	28 (43.1%)	
	Asian	2 (1.8%)	0 (0.0%)	
	Other	0 (0.0%)	1 (1.5%)	
**CV1, DEM_ Current married/partner status**				
	Never married	24 (21.2%)	23 (35.4%)	**0.034**
	Divorced	0 (0.0%)	2 (3.1%)	
	Separated	5 (4.4%)	0 (0.0%)	
	Living with partner	10 (8.8%)	5 (7.7%)	
	Married	71 (62.8%)	34 (52.3%)	
	Missing	3 (2.7%)	1 (1.5%)	
**CV1, DEM_ Health insurance coverage (private/other)**				
	Public	34 (30.1%)	31 (47.7%)	**0.034**
	Private	75 (66.4%)	33 (50.8%)	
	Missing	4 (3.5%)	1 (1.5%)	
**CV1, DEM_ Estimated total annual household income**				
	<25K	31 (27.4%)	28 (43.1%)	0.088
	25-65K	42 (37.2%)	18 (27.7%)	
	> 65K	37 (32.7%)	16 (24.6%)	
	Missing	3 (2.7%)	3 (4.6%)	
Mother				
**M2, TLEQ_ Count of Adverse Child Events**		0.4 (±0.8)	0.7 (±0.9)	**0.009**
	Missing	3 (2.7%)	2 (3.1%)	
**M2, CTS_ Frequency of Physical Aggression Score Self**		2.3 (±3.7)	3.6 (±4.1)	**0.015**
	Missing	3 (2.7%)	2 (3.1%)	
**CV1, CAPI_ Rigidity Scale Total Score**		12.7 (±11.7)	18.2 (±14.7)	**0.022**
	Missing	5 (4.4%)	1 (1.5%)	
**CV1, CAPI_ Abuse Scale Total Score**		62.1 (±50.9)	89.7 (±80.5)	**0.047**
	Missing	5 (4.4%)	1 (1.5%)	

**HV1, Pregnant or breastfeeding**			
No	32 (28.3%)	27 (41.5%)	0.092
Yes	74 (65.5%)	34 (52.3%)	
Missing	7 (6.2%)	4 (6.2%)	
**HV1, History of alcohol consumption**			
No	50 (44.2%)	20 (30.8%)	0.076
Yes	56 (49.6%)	41 (63.1%)	
Missing	7 (6.2%)	4 (6.2%)	
**M1, TEMPS_ Hyperthymic temperament**			
No	37 (32.7%)	31 (47.7%)	0.056
Yes	76 (67.3%)	34 (52.3%)	
Child			
**M3, NSF_ Birth length**	51.2 (±2.5)	50.2 (±2.6)	**0.028**
Missing	1 (0.9%)	1 (1.5%)	

CV2	Class 2B2 n=113	Class 2B1 n=65	P-value
Family			
**M1, DEM_ Race**			
Black	33 (29.2%)	36 (55.4%)	**0.0005**
White	78 (69.0%)	28 (43.1%)	
Asian	2 (1.8%)	0 (0.0%)	
Other	0 (0.0%)	1 (1.5%)	
**CV2, DEM_ Estimated total annual household income**			
<25K	29 (25.7%)	28 (43.1%)	0.062
25-65K	39 (34.5%)	19 (29.2%)	
>65K	40 (35.4%)	16 (24.6%)	
Missing	5 (4.4%)	2 (3.1%)	
**CV2, DEM_ Health insurance coverage (private/other)**			
Public	40 (35.4%)	32 (49.2%)	0.079
Private	73 (64.6%)	32 (49.2%)	
Missing	0 (0.0%)	1 (1.5%)	
Maternal			
**M2, TLEQ_ Count of Adverse Child Events**	0.4 (±0.8)	0.7 (±0.9)	**0.009**
Missing	3 (2.7%)	2 (3.1%)	
**CV2, CAPI_ Abuse Scale Total Score**	65.9 (±67.1)	85.7 (±82.2)	0.08
Missing	0 (0%)	1 (1.5%)	
**CV1, BSI_ T-score for Somatization scale**	46.8 (±7.9)	49.3 (±9.0)	0.069
Missing	5 (4.4%)	1 (1.5%)	

**HV1, History of alcohol consumption**				
	Yes	50 (44.2%)	20 (30.8%)	**0.076**
	No	56 (49.6%)	41 (63.1%)	
	Missing	7 (6.2%)	4 (6.2%)	
**HV1, Pregnant or breastfeeding**				
	No	32 (28.3%)	27 (41.5%)	**0.092**
	Yes	74 (65.5%)	34 (52.3%)	
	Missing	7 (6.2%)	4 (6.2%)	
Child				
**CV2, BITSEA_ Internalizing subscale, subcomponent of Problem scale**		1.8 (±1.5)	2.5 (±1.7)	**0.008**
	Missing	0 (0%)	1 (1.5%)	
**M3, NSF_ Birth length (cm)**		51.2 (±2.5)	50.2 (±2.6)	**0.028**
	Missing	1 (0.9%)	1 (1.5%)	

CV3		Class 2B2<br>n=113	Class 2B1<br>n=65	P-value
Family				
**M1, DEM_ Race**				
	Black	33 (29.2%)	36 (55.4%)	**0.0005**
	White	78 (69.0%)	28 (43.1%)	
	Asian	2 (1.8%)	0 (0.0%)	
	Other	0 (0.0%)	1 (1.5%)	
Mother				
**CV3, CAPI_ Unhappiness Scale Total Score**		8.4 (±8.0)	12.4 (±11.9)	**0.026**
	Missing	6 (5.3%)	2 (3.1%)	
**CV3, CAPI_ Abuse Scale Total Score**		62.7 (±60.6)	86.6 (±81.8)	**0.035**
	Missing	6 (5.3%)	2 (3.1%)	
**CV3, CAPI_ Rigidity Scale Total Score**		12.1 (±11.9)	17.3 (±15.4)	**0.039**
	Missing	6 (5.3%)	2 (3.1%)	
**HV1, History of alcohol consumption**				
	1	50 (44.2%)	20 (30.8%)	0.076
	2	56 (49.6%)	41 (63.1%)	
	Missing	7 (6.2%)	4 (6.2%)	
**HV1, Pregnant or breastfeeding**				
	1	32 (28.3%)	27 (41.5%)	0.092
	2	74 (65.5%)	34 (52.3%)	
	Missing	7 (6.2%)	4 (6.2%)	

Child			
**CV3, CBCL_ Anxiety Problems %tile Rank**	57.4 (±13.7)	60.3 (±13.6)	**0.022**
Missing	7 (6.2%)	2 (3.1%)	
**M3, NSF_ Birth length (cm)**	51.2 (±2.5)	50.2 (±2.6)	**0.028**
Missing	1 (0.9%)	1 (1.5%)	
**CV3, CBCL_ Affective Problems %tile Rank**	60.8 (±12.7)	56.4 (±9.4)	**0.08**
Missing	7 (6.2%)	2 (3.1%)	
