# Supplementary material for: Longitudinal Trajectories of Hair Cortisol: Hypothalamic-Pituitary-Adrenal Axis Dysfunction in Early Childhood
Source: Front Pediatr. 2021 Oct 11;9:740343. doi: 10.3389/fped.2021.740343 (PMC8544285; doi:10.3389/fped.2021.740343)
Supplement: Supplementary file 8 [file Data_Sheet_8.PDF]

Supplementary Table G: STROBE Statement—checklist for reports of observational studies

	Item	Recommendation	Reported?	Page#
**Title and abstract**	1	(*a*) Indicate the study's design with a commonly used term in the title or the abstract	Yes	1
		(*b*) Provide in the abstract an informative and balanced summary of what was done and what was found	Yes	1-2
**Introduction**				
Background/rationale	2	Explain the scientific background and rationale for the investigation being reported	Yes	2-3
Objectives	3	State specific objectives, including any prespecified hypotheses	Yes	2-3
**Methods**				
Study design	4	Present key elements of study design early in the paper	Yes	3-4
Setting	5	Describe the setting, locations, and relevant dates, including periods of recruitment, exposure, follow-up, and data collection	Yes	3
Participants	6	(*a*) *Cohort study*—Give the eligibility criteria, and the sources and methods of selection of participants. Describe methods of follow-up<br>*Case-control study*—Give the eligibility criteria, and the sources and methods of case ascertainment and control selection. Give the rationale for the choice of cases and controls<br>*Cross-sectional study*—Give the eligibility criteria, and the sources and methods of selection of participants	Yes, cohort	3
		(*b*) *Cohort study*—For matched studies, give matching criteria and number of exposed and unexposed<br>*Case-control study*—For matched studies, give matching criteria and the number of controls per case	N/A	
Variables	7	Clearly define all outcomes, exposures, predictors, potential confounders, and effect modifiers. Give diagnostic criteria, if applicable	Yes	4
Data sources/ measurement	8*	For each variable of interest, give sources of data and details of methods of assessment (measurement). Describe comparability of assessment methods if there is more than one group	Yes	4

Bias	9	Describe any efforts to address potential sources of bias	Yes	5
Study size	10	Explain how the study size was arrived at	Yes	5
Quantitative variables	11	Explain how quantitative variables were handled in the analyses. If applicable, describe which groupings were chosen and why	Yes	3-4
Statistical methods	12	(*a*) Describe all statistical methods, including those used to control for confounding	Yes	3-4
		(*b*) Describe any methods used to examine subgroups and interactions	Yes	3-4
		(*c*) Explain how missing data were addressed	Yes	3-4
		(*d*) *Cohort study*—If applicable, explain how loss to follow-up was addressed *Case-control study*—If applicable, explain how matching of cases and controls was addressed *Cross-sectional study*—If applicable, describe analytical methods taking account of sampling strategy	Yes	6
		(*e*) Describe any sensitivity analyses	N/A	

**Results**			**Reported?**	**Page#**
Participants	13*	(a) Report numbers of individuals at each stage of study—eg numbers potentially eligible, examined for eligibility, confirmed eligible, included in the study, completing follow-up, and analysed	Yes	1, 4-5
		(b) Give reasons for non-participation at each stage	Yes	3
		(c) Consider use of a flow diagram	N/A	
Descriptive data	14*	(a) Give characteristics of study participants (eg demographic, clinical, social) and information on exposures and potential confounders	Yes	7-9
		(b) Indicate number of participants with missing data for each variable of interest	Yes	Supp Tables A-F
		(c) *Cohort study*—Summarise follow-up time (eg, average and total amount)	Yes	1, 3
Outcome data	15*	*Cohort study*—Report numbers of outcome events or summary measures over time	Yes	Supp Table B, 4
		Case-control study—Report numbers in each exposure category, or summary measures of exposure	N/A	

		Cross-sectional study—Report numbers of outcome events or summary measures	N/A	
Main results	16	(*a*) Give unadjusted estimates and, if applicable, confounder-adjusted estimates and their precision (eg, 95% confidence interval). Make clear which confounders were adjusted for and why they were included	Yes	Table 1
		(*b*) Report category boundaries when continuous variables were categorized	Yes	4-5
		(*c*) If relevant, consider translating estimates of relative risk into absolute risk for a meaningful time period	N/A	
Other analyses	17	Report other analyses done—eg analyses of subgroups and interactions, and sensitivity analyses	Yes	4-5. Supp Tables E-H3
**Discussion**				
Key results	18	Summarise key results with reference to study objectives	Yes	5-7
Limitations	19	Discuss limitations of the study, taking into account sources of potential bias or imprecision. Discuss both direction and magnitude of any potential bias	Yes	7
Interpretation	20	Give a cautious overall interpretation of results considering objectives, limitations, multiplicity of analyses, results from similar studies, and other relevant evidence	Yes	5-7
Generalisability	21	Discuss the generalisability (external validity) of the study results	Yes	7
**Other information**				
Funding	22	Give the source of funding and the role of the funders for the present study and, if applicable, for the original study on which the present article is based	Yes	8
