# Supplementary material for: Longitudinal Trajectories of Hair Cortisol: Hypothalamic-Pituitary-Adrenal Axis Dysfunction in Early Childhood
Source: Front Pediatr. 2021 Oct 11;9:740343. doi: 10.3389/fped.2021.740343 (PMC8544285; doi:10.3389/fped.2021.740343)
Supplement: Supplementary file 9 [file Data_Sheet_9.PDF]

Supplementary Table H: Checklist for Reporting on Latent Trajectory Studies (GRoLTS)

	Item	Reported ?	Page#
1	Is the metric of time used in the statistical model reported?	Yes	1, 3-5
2	Is information presented about the mean and variance of time within a wave?	Yes	4-5
3a	Is the missing data mechanism reported?	Yes	Supp Table B, 2, 7
3b	Is a description provided of what variables are related to attrition/missing data?	Yes	Supp Tables A & B
3c	Is a description provided of how missing data in the analyses were dealt with?	Yes	2, 7
4	Is information about the distribution of the observed variables included?	Yes	Supp Tables D, E, F
5	Is the software mentioned? Yes/No	Yes	4
6a	Are alternative specifications of within-class heterogeneity considered (e.g., LGCA vs. LGMM) and clearly documented? If not, was sufficient justification provided as to eliminate certain specifications from consideration?	No	
6b	Are alternative specifications of the between-class differences in variance–covariance matrix structure considered and clearly documented? If not, was sufficient justification provided as to eliminate certain specifications from consideration?	No	
7	Are alternative shape/functional forms of the trajectories described?	No	
8	If covariates have been used, can analyses still be replicated?	Yes	
9	Is information reported about the number of random start values and final iterations included?	No	
10	Are the model comparison (and selection) tools described from a statistical perspective?	Yes	3-4
11	Are the total number of fitted models reported, including a one-class solution?	Yes	1-5
12	Are the number of cases per class reported for each model (absolute sample size, or proportion)?	Yes	4-5
13	If classification of cases in a trajectory is the goal, is entropy reported?	No	BIC, Supp Table C
14a	Is a plot included with the estimated mean trajectories of the final solution?	Yes	Figures 1-4
14b	Are plots included with the estimated mean trajectories for each model?	Yes	Figures 1-4
14c	Is a plot included of the combination of estimated means of the final model and the observed individual trajectories split out for each latent class?	Yes	Figures 1-4
15	Are characteristics of the final class solution numerically described (i.e., means, SD/SE, n, CI, etc.)?	Yes	Figure 4
16	Are the syntax files available (either in the appendix, supplementary materials, or from the authors)?	Yes	On request

Note. LGCA = latent class growth analysis; LGMM = latent growth mixture modeling.
