# Supplementary material for: Longitudinal Trajectories of Hair Cortisol: Hypothalamic-Pituitary-Adrenal Axis Dysfunction in Early Childhood
Source: Front Pediatr. 2021 Oct 11;9:740343. doi: 10.3389/fped.2021.740343 (PMC8544285; doi:10.3389/fped.2021.740343)
Supplement: Supplementary file 10 [file Image_1.pdf]

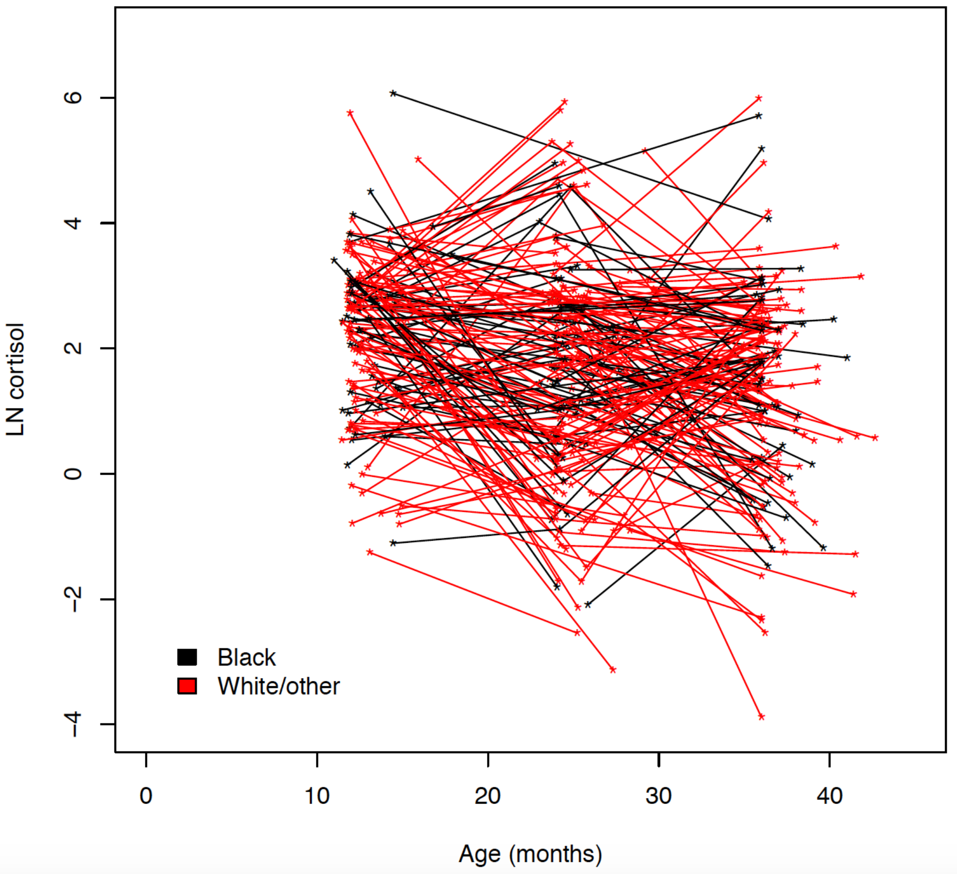

Supplimental Figure 1: Longitudinal plot of log-natural hair cortisol concentrations (ln-HCC) in 265 children plotted by age in months; color-coded lines for race with Blacks (black) and White/other in red.
